# Seasonal characteristics of influenza vary regionally across US

**DOI:** 10.1371/journal.pone.0212511

**Published:** 2019-03-06

**Authors:** James Tamerius, Christopher Uejio, Jeffrey Koss

**Affiliations:** 1 University of Iowa, Iowa City, Iowa, United States of America; 2 Florida State University, Tallahassee, Florida, United States of America; Columbia University, UNITED STATES

## Abstract

Given substantial regional differences in absolute humidity across the US and our understanding of the relationship between absolute humidity and influenza, we may expect important differences in regional seasonal influenza activity. Here, we assessed cross-seasonal influenza activity by comparing counts of positive influenza A and B rapid test results during the influenza season versus summer baseline periods for the 2016/2017 and 2017/2018 influenza years. Our analysis indicates significant regional patterns in cross-seasonal influenza activity, with relatively fewer influenza cases during the influenza season compared to summertime baseline periods in humid areas of the US, particularly in Florida and Hawaii. The cross-seasonal ratios vary from year-to-year and influenza type, but the geographic patterning of the ratios is relatively consistent. Mixed-effects regression models indicated absolute humidity during the influenza season was the strongest predictor of cross-seasonal influenza activity, suggesting a relationship between absolute humidity and cross-seasonal influenza activity. There was also evidence that absolute humidity during the summer plays a role, as well. This analysis suggests that spatial variation in seasonal absolute humidity levels may generate important regional differences in seasonal influenza activity and dynamics in the US.

## Introduction

Improving our understanding of the seasonal nature of influenza is important since it provides insight into the underlying mechanisms modulating influenza transmission [1–3], it likely has important effects on viral evolutionary processes [4–6], and because it can illuminate pandemic influenza patterns [7–11]. As such, numerous studies have investigated seasonal influenza epidemic patterns and have shown that influenza is strongly seasonal in temperate regions with epidemics that peak during the winter; whereas in subtropical and tropical regions the seasonality of influenza can be less pronounced, characterized by multiple peaks, or relatively aseasonal [1, 12–16].

A non-linear relationship between absolute humidity and influenza activity has been suggested to underlie the seasonal influenza signals observed globally. Although some laboratory studies have found no measurable relationship between influenza and humidity [17], most laboratory studies indicate that low absolute humidity increases the survival and transmission of influenza [3, 18] which is consistent with annual influenza epidemics that peak during the winter when absolute humidity is near minimal levels in temperate regions [3, 19–21]. On the other hand, in subtropical and tropical regions influenza activity tends to peak when absolute humidity is at high levels [16, 19, 22, 23]. Although the associations between influenza and absolute humidity for temperate and tropical regions appear paradoxical, these observations have been reconciled by studies showing a U-shaped relationship between influenza transmission and absolute humidity, with suppressed influenza activity when absolute humidity is at moderate levels [19, 23]. That said, the mechanisms that link absolute humidity and transmission are not fully understood, and it is possible that additional and confounding factors are responsible for the relationships observed.

A recent study showed that Australia is characterized by wintertime influenza epidemics, but summer influenza activity is elevated in the northern subtropical and tropical regions of the country [5]. The study also used phylogenetic data to show that many viruses circulating in the summer displayed extended chains of transmission that continued into the winter influenza season, suggesting that the increased summer influenza activity in tropical and subtropical Australia may play an important role in seasonal influenza dynamics. Given the significant spatial and seasonal variation in absolute humidity in the US, and our current understanding of the relationship between humidity and influenza activity, it would not be surprising if the seasonal nature of influenza varies across the US which may impact seasonal influenza dynamics.

As such, here we investigate spatial differences in influenza activity during the influenza season relative to activity outside of the influenza season, or “cross-seasonal influenza activity.” We use a recently available influenza dataset that is highly resolved in both spatial and temporal dimensions and, more importantly, distinguishes between influenza A and B. These detailed influenza A and B time-series allow us to show significant regional differences in the seasonal nature of influenza in the US, and develop hypotheses about their causes.

## Methods

### Data

We received influenza A and B test results for July 1, 2016—June 30, 2018 from the Quidel Corporation. These tests results are generated by a network of diagnostic sensors based at clinics, hospitals and pharmacies through the Virena platform. The Virena platform uses an immunofluorescence-based, lateral-flow assay that automatically transmit the data in real-time to a cloud based database. The HIPPA compliant data are de-identified and include the location (zip code), result (positive/negative), unique identifier for the reporting sensor, and date of each test performed. The analysis included results from sensors that reported 1 or more results in the prior year and in the final 90 days of the influenza year being analyzed.

We aggregated the influenza data from each sensor into geographic “subregions” that are composed of groups of metropolitan areas that share economic, cultural and environmental characteristics. The subregions were previously defined by the strength of commuter traffic flows [24]. S1 Fig. maps the states that comprise each subregion and the distribution of reporting sensors. We also added Honolulu, HI as a unique subregion. Aggregating the data across these broader spatial units (e.g., versus zip codes or metropolitan areas) increased the number of test results per spatial unit, improving the stability of our measures of cross-seasonal influenza activity (see below).

Specific humidity (a measure of absolute humidity, or total amount of moisture in the air) and air temperature were obtained from the NCEP/NCAR Global Reanalysis (GR) dataset for 2016–2018 [25]. The temperature and humidity data was extracted from the GR for the centroid of the largest metropolitan area in each subregion. The GR was used because it provides up-to-date environmental data across the US. The drawback of the GR dataset is its coarse spatial scale (2º x 2º latitude/longitude), which can introduce statistical bias to humidity and temperature estimates. Specific humidity and temperature were weighted by the number of positive influenza tests to better reflect the average conditions during influenza transmission. S2 Fig. illustrates the weighted average and maximum or minimum of specific humidity for the baseline and influenza seasons.

The population size of each subregion was derived from census tract level population counts from the 2010 US Census [26]. Vaccination rates were calculated using state level vaccination data from the Center for Disease Control and Prevention for the 2016–2017 season [27]. For subregions that intersected multiple states, a weighted average of the vaccination rate was calculated based upon the contribution of each state to the total subregion population.

### Analysis

A straightforward way to measure spatial variation in seasonal influenza activity would be to calculate attack rates (i.e., the proportion of individuals infected), and compare across subregions and seasons. However, since the data did not allow us to estimate total number of infected individuals in each subregion we could not estimate attack rates, instead, we calculated a relative measure of influenza activity by comparing counts of positive influenza results between “influenza” and “baseline” seasons. For each analytical procedure, we assessed the 2016–2017 and the 2017–2018 “influenza years” (July 1—June 30) independently.

### Defining the influenza and baseline seasons

We defined the influenza season as the 270-day (~9 months) window corresponding to the maximum count of positive tests during an influenza year. The baseline season was defined as the remaining 95-days outside of the influenza season. These seasons were defined independently for each subregion, influenza type and year to mitigate the potential impacts of differential timing of the influenza/baseline seasons on the analysis. Given the window size arbitrarily defines the duration of the influenza and baseline season, we assessed, as a sensitivity analysis, the consistency of the results for influenza seasons defined as the window corresponding to the maximum count of positive tests for 180 and 330 days.

A “cross-seasonal ratio” was defined as the number of positive influenza tests during the influenza season divided by the number of positive tests during the baseline season. Subregions with fewer than 170 total positive tests during the influenza year were dropped from the analysis. Although aggregating the data to the subregional level increased the number of cases in each spatial unit, we still had subregions with limited data. As such, we artificially added a single positive-test to the baseline season for subregions that had over 170 positive tests, but no positive tests during the baseline season, to avoid division by zero. As additional support for our findings, we also calculated the influenza percent-positivity rate, i.e., the percent of tests that were positive for influenza, for both the epidemic and baseline seasons.

We used multiple Fisher’s exact tests to evaluate if the observed frequencies of influenza cases during the influenza and baseline seasons in each subregion significantly diverged from expected frequencies based on the data pooled across all other subregions. We used the Benjamini-Hochberg procedure to correct for the multiple tests performed, with the false discovery rate (Q) set to 0.01 [28]. We then assessed spatial autocorrelation of the cross-seasonal ratios (log transformed) using global Moran’s I (MI) [29]. Neighbors were defined as subregions whose centroid fell within a 600 km radius of each subregion. This threshold distance generally included 4–7 of a subregion’s nearest neighbors. The spatial weights matrix was row standardized and the MI were calculated using the PySAL library [30]. Likewise, we used Fisher’s Exact tests to evaluate differences in percent-positivity for each subregion as compared against the data pooled from all other subregions, and corrected for multiple testing using the Benjamini-Hochberg procedure.

Next, we assessed the relationship between the cross-seasonal ratios and candidate independent variables using bivariate mixed-effects maximum likelihood regression. The dependent variable was the log of cross-seasonal ratios (it was transformed to reduce the weight of outliers). Different models considered each independent variable: seasonal (epidemic or baseline) weighted-mean specific humidity or temperature (we also included square humidity and temperature terms to assess non-linear relationships), latitude, longitude, vaccination rate, or total population. We treated each subregion as a random effect to account for the repeated samples from each unit. After selecting the best fitting bivariate relationships, we further adjusted the effect estimates by including fixed effect dummy variables to account for year and influenza type. Finally, a forward stepwise AIC variable selection procedure tested whether the best fitting bivariate relationship could be further improved by the remaining independent variables.

## Results

**Fig 1:**
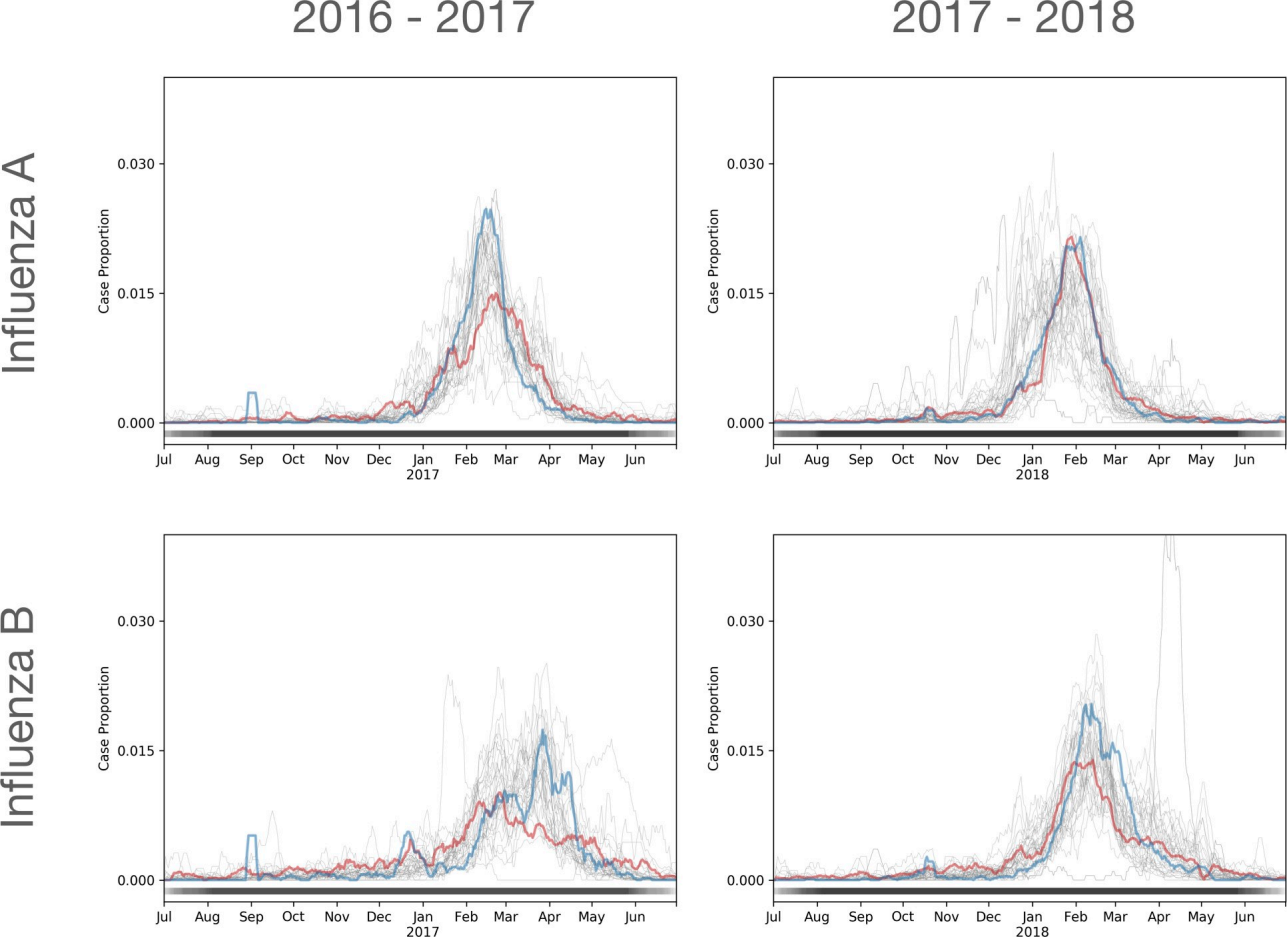
7-day moving average of positive test results for influenza A and B in each subregion. The y-axis indicates the proportion of annual positive test results. To highlight differences is seasonal characteristics across regions we display Orlando, FL (red) and Des Moines, IA (blue). The thick horizontal line at the bottom of the plot shows the distribution of the beginning/end of the influenza season; black areas are within the influenza season for all locations and lighter shades are a mix between influenza and baseline seasons.

We analyzed the results of 755,170 tests from 7,533 unique sensors administered between July 1, 2016 and June 30, 2018, with 136,793 positive for influenza A and 86,498 positive for influenza B (S1 Fig). The number of subregions analyzed varied between 34–40 by year and influenza type (Tables 1 and 2). In general, the 270-day window with the greatest influenza activity was from early September through the end of May, with influenza A and B influenza seasons highly aligned (Fig 1). Accordingly, the summer baseline season was defined as approximately June-August for a wide majority of subregions in both years and influenza types. Median cross-seasonal ratios varied by influenza type and year, ranging from 104–426 (Table 1).

**Table 1:** Basic summary of the results stratified by year and influenza type. Moran’s I values are similar to correlation coefficients with values typically ranging from -1 to 1 and higher values indicating higher spatial autocorrelation.

Type / Influenza Year	# Subregions	# Positive Influenza Season	# Positive Baseline Season	Median Cross-Seasonal Ratio	Moran’s I
Influenza A / 2016–2017	37	57,554	386	237	0.24 (p<0.01)
Influenza A / 2017–2018	40	79,239	547	426	0.19 (p<0.02)
Influenza B / 2016–2017	34	37,521	632	104	0.42 (p<0.001)
Influenza B / 2017–2018	37	48,977	613	277	0.30 (p<0.001)

**Table 2:** Cross-seasonal ratios for each subregion by year and influenza type. For more detailed results see the S1 File.

	2016 / 2017		2017 / 2018	
Subregion	Influenza A	Influenza B	Influenza A	Influenza B
Albany	—	—	1189	513
Albuquerque	283	—	740	535
Atlanta	132	77	699	133
Austin	51	28	257	131
Birmingham	33	18	264	89
Boston	211	159	536	191
Buffalo	606	393	454	411
Charleston	119	21	338	54
Charlotte	61	34	1410	131
Chicago	178	184	592	753
Cleveland	—	—	421	—
Columbus	598	413	605	316
Corpus Christi	299	99	437	150
Dallas	148	46	671	232
Denver	543	109	409	231
Des Moines	1730	331	2535	1586
Detroit	780	150	1653	836
Honolulu	16	10	59	33
Houston	105	29	470	212
Indianapolis	—	—	410	212
Kansas City	400	226	684	375
Knoxville	546	468	655	163
Louisville	114	33	1642	1301
Lubbock	253	41	3299	805
Memphis	122	25	945	2615
Miami	—	—	58	39
Midland	258	100	1072	527
Milwaukee	289	506	337	257
Minneapolis	388	430	2555	755
Nashville	105	15	632	339
New Orleans	162	44	411	151
New York City	109	—	1639	1627
Oklahoma City	357	101	821	5313
Omaha	237	397	506	1998
Orlando	54	19	158	70
Phoenix	247	257	486	105
Pittsburgh	—	—	259	—
Raleigh	381	135	1304	771
Richmond	150	105	396	410
San Diego	—	—	205	169
San Francisco	—	—	163	—
Seattle	—	—	442	434
Sioux Falls	239	221	270	—
St Louis	153	—	205	—
Washington DC	1363	122	1098	666

**Fig 2:**
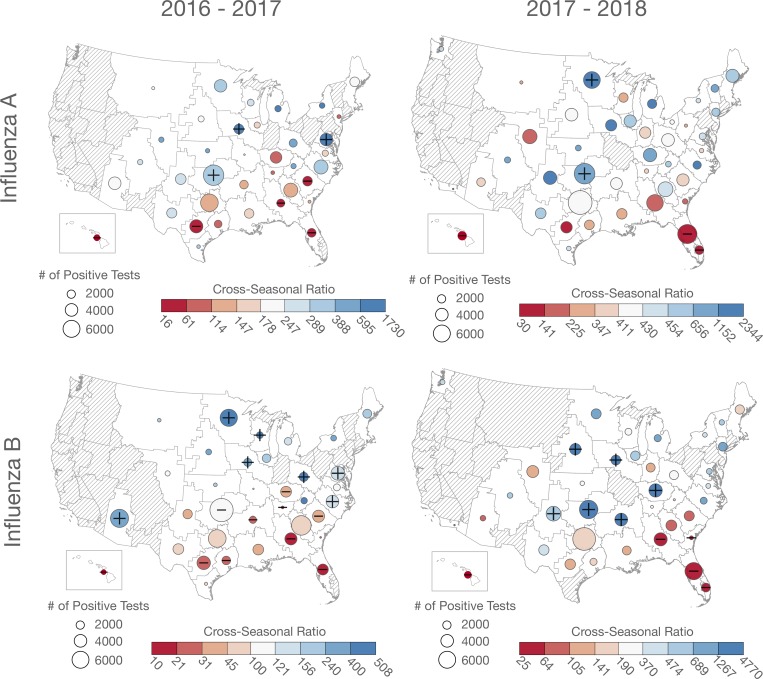
Maps of cross-seasonal ratios for each subregion by influenza year and type. Plus/minus symbols indicate subregions where the ratio was significantly below/above expected value (based on Fisher’s exact tests). Dashed areas are subregions with no or inadequate numbers of positive tests.

**Fig 3:**
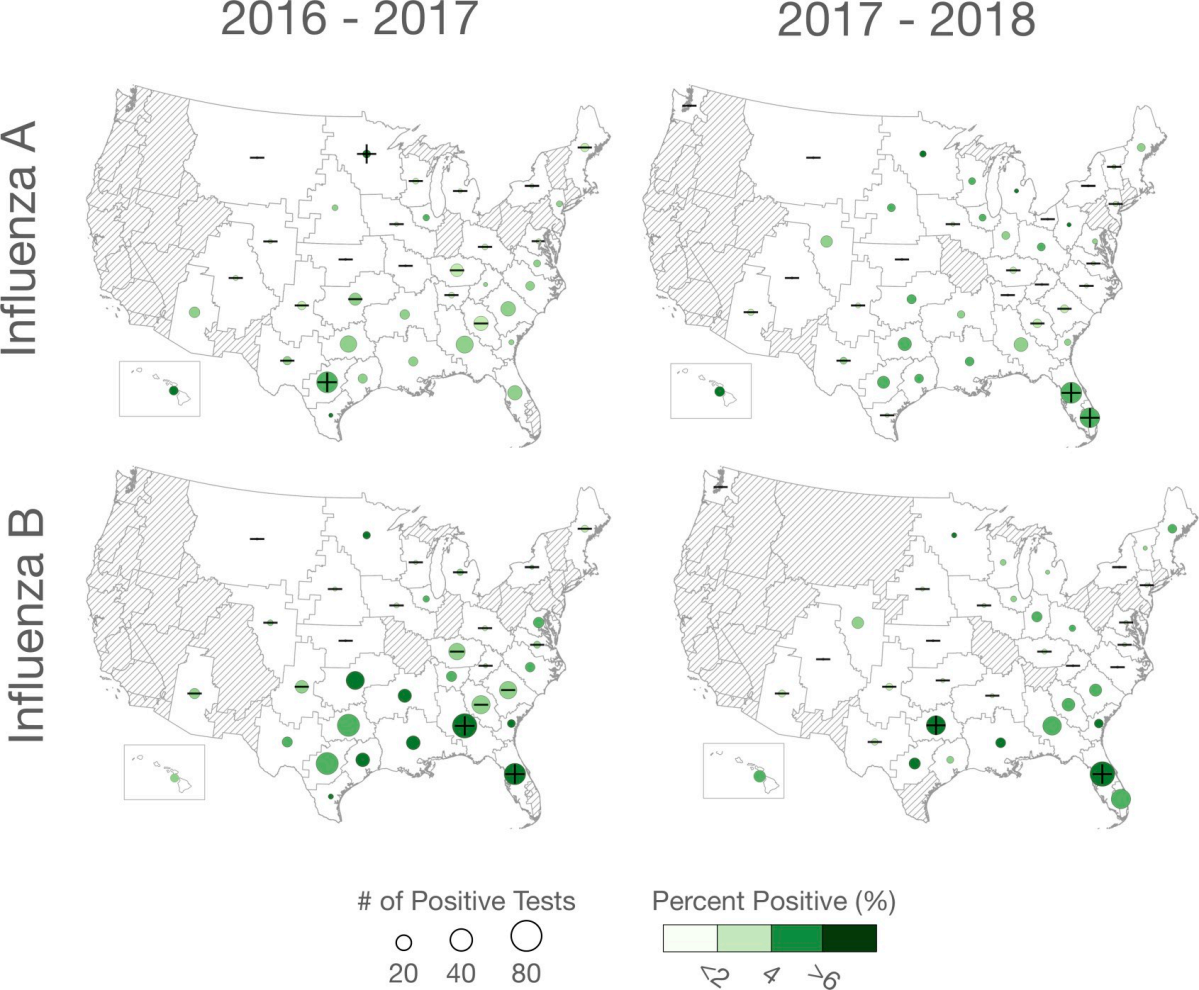
Percent-positivity rates during the baseline season for influenza A and B during 2016–2017 and 2017–2018. Plus/minus symbols indicate subregions where the ratio was significantly below/above expected value (based on Fisher’s exact tests). Dashed areas are subregions with no or inadequate numbers of positive tests.

The cross-seasonal ratios were spatially autocorrelated for each influenza type and year, and the autocorrelation tended to be stronger for influenza B than for influenza A (Table 1). The spatial patterns were characterized by lower cross-seasonal ratios in the southeastern quadrant of the US and Hawaii (HI), and they were always significantly lower (p<0.0001, Q<0.01) in Florida (FL) and HI (Table 2, Figs 2 and 3). Outside of the southeastern US and HI, there tended to be little or no trend in cross-seasonal ratios (Fig 2). Although the general spatial structure of the cross-seasonal ratio patterns observed was consistent across types and years, the pattern was weakest during the severe 2017–2018 influenza A season (Table 1, Fig 2 and S1 File). The geographic pattern of the ratios was relatively consistent across all seasonal definitions, with lower ratios in the southeastern US and HI (S2 Fig).

We also assessed the percent-positivity rate for each year and influenza type. During the influenza season positivity rates averaged approximately 14–19% and 7–13% for influenza A and B, respectively. There did not appear to be a consistent geographic pattern to positivity rates during the influenza season (S3 Fig). During the summer baseline season influenza positivity rates were lower, averaging 2–5% and 1–4% for influenza A and B, respectively (Fig 3). There was a strong geographic trend in positivity rates during the summer baseline season with rates significantly lower outside of the southeastern US, and a higher number of positive tests for influenza A and B in the southeastern US (Fig 3).

### Mixed-effects models

Bivariate mixed-effects regression analysis showed several variables were significantly associated with cross-seasonal ratios, including temperature during the influenza season, specific humidity during the baseline season, latitude and vaccination rate (Table 3). Based on BIC, however, weighted specific humidity during the influenza season was a stronger fit than the other variables (AIC only provided marginal support over other models). Adding dummy variables that accounted for differences in influenza type and year also significantly improved the fit (Table 4). Adding additional variables to the model containing these dummy variables and specific humidity (during influenza season) did not improve the model. Moran’s I showed that the residuals from the mixed effects models were not spatially autocorrelated (Moran’s I = 0.01, p-value = 0.48).

**Table 3:** Results of bivariate mixed-effects regression analysis where the log of the cross-seasonal ratio was the dependent variable. The models were sorted in ascending order by AIC.

Predictors	Bivariate	
	Coefficients (95% CI)	AIC/BIC
Weighted specific humidity (influenza season)	-0.14 (-0.16, -0.11)	145/157
Weighted temperature (influenza season)	-0.04 (-.05, -.04)	161/173
Latitude	0.04 (0.03, 0.06)	198/210
Weighted specific humidity (baseline season)	-0.05 (-0.07, -0.03)	208/220
Vaccination Rate	0.05 (0.02, 0.08)	213/225
Weighted temperature (baseline season)	-0.02 (-0.04, 0.00)	221/233
Longitude	0.00 (-0.01, 0.01)	225/237
Total Population / 10^5^	0.01 (-0.26, 0.28)	225/237

**Table 4:** Results of select multivariate mixed-effects regression models where the log cross-seasonal ratio was the dependent variable. The models were sorted in ascending order by AIC. A null model that only includes the dummy variables was included for comparison.

	Model 1		Model 2		Model 3		Null Model	
Predictors	Coefficients (95% CI)	AIC/BIC	Coefficients (95% CI)	AIC/BIC	Coefficients (95% CI)	AIC/BIC	Coefficients (95% CI)	AIC/BIC
Latitude	—	105/126	—	107/131	0.00 (-0.02, 0.02)	107/132	—	146/164
Longitude	—		—		—		—	
Weighted specific humidity (influenza season)	-0.10 (-0.13, -0.08)		-0.10 (-0.13, -0.07)		-0.10 (-0.14, -0.06)		—	
Weighted specific humidity (baseline season)	—		0.00 (-0.02, 0.02)		—		—	
Weighted temperature (influenza season)	—		—		—		—	
Weighted temperature (baseline season)	—		—		—		—	
Total Population	—		—		—		—	
Vaccination Rate	—		—		—		—	
Influenza A/2016-2017	-0.15 (-0.28, -0.01)		-0.15 (-0.28, -0.01)		-0.15 (-0.29, -0.01)		-0.26 (-0.39, -0.12)	
Influenza A/2017-2018	0.14 (0.01, 0.27)		0.14 (0.01, 0.27)		0.14 (0.01, 0.27)		0.21 (0.07, 0.34)	
Influenza B/2016-2017	-0.40 (-0.55, -0.26)		-0.40 (-0.55, -0.25)		-0.40 (-0.56, -0.25)		-0.60 (-0.74, -0.46)	
Influenza B/2017-2018	0		0		0		0	

As a sensitivity analysis, we also defined the influenza season as the 180 and 330 days with maximum influenza counts. For both alternative definitions of the influenza season, the mixed-effects regression models showed that specific humidity was the strongest predictor of cross-seasonal ratios (S1 Table and S2 Table). Indeed, the multivariate models for the 180 day influenza season were similar to the main results (S3 Table). However, when the influenza season was defined by 330 days, specific humidity during the baseline season–rather than the influenza season–generated the best bivariate model. Further, based on AIC (but not BIC), the combination of specific humidity during the influenza and baseline seasons provided the best multivariate model fit, although this was only a marginal improvement relative to other models with baseline specific humidity and baseline temperature (S4 Table).

## Discussion

Here we measured variation in the seasonal distribution of influenza activity across the US by contrasting influenza activity within and outside of the influenza season, or “cross-seasonal” influenza activity. The results indicate that seasonal characteristics of influenza vary across the US, and that this variation is associated with absolute humidity. We suggest that these seasonal changes may highlight important differences in seasonal influenza dynamics in the US.

Specifically, our analysis suggests that seasonal characteristics of influenza vary across the US, following climatological gradients (S4 Fig). We analyzed two years of influenza data for ~40 subregions and found that the rate of influenza detection during the influenza season relative to the summer season to be lower in the southeastern US and HI (Table 2, Fig 2). For example, during the 2016–2017 influenza season the median cross-seasonal ratio in the southeastern US and HI was approximately 80/1 for influenza A, meaning for every positive influenza A test result in the influenza season, there was one positive influenza A test result in the summer baseline season; whereas it was 280/1 outside of these regions (Table 2). The spatial patterning of cross-seasonal ratios was broadly consistent for influenza A and B in both years with lower ratios in the southeastern quadrant of the US and HI (Fig 2), however, the ratios varied by year and influenza type (Table 1).

Cross-seasonal ratios provided significant analytical traction for detecting differences in the seasonal characteristics of influenza across the US, however, this measure was not well suited for determining whether the differences observed were associated with processes during the influenza and/or the summer baseline season. However, our mixed-effects regression models showed that absolute humidity levels during the influenza season demonstrated a much stronger fit to the data than other variables assessed (Tables 3 and 4), suggesting that the underlying mechanism causing variation in cross-seasonal ratios is related to humidity during the influenza season. Given the moderate levels of absolute humidity during the influenza season in the southeastern US and HI (S4 Fig), this is consistent with our understanding of absolute humidity and influenza, i.e., influenza transmission is less efficient in moderate levels of humidity. This also aligns with the result of a past study showing a significant and negative relationship between influenza mortality and absolute humidity (during the influenza season) across the US [31]. Overall, this suggests that the differences in cross-seasonal ratios may be, at least partially, a result of decreases in influenza activity during the influenza season in humid regions.

We also observed increased influenza percent-positivity rates during the summer baseline season in the southeastern US across both years and subtypes, and there were more positive test results in the southeastern US during the baseline season than in other regions (Fig 3). This suggests that influenza activity is greater in the southeastern US and HI during the humid summer season. Further, when we extended the influenza season to 330 days, specific humidity during the baseline season (i.e., the summer) provided the best-fit to cross-seasonal ratios, which is consistent with the results of previous studies showing increased influenza activity in some subtropical regions when absolute humidity is at high levels [16, 19, 22, 23].

Altogether, our analysis suggests that variability of cross-seasonal ratios may be driven by spatial variability in the intensity of influenza activity during both the influenza and summer baseline seasons. A potential mechanistic explanation of our findings is that the moderate-humidity levels that characterize the southeastern US and HI during the influenza season may slightly suppress influenza activity (i.e., lower attack rates), which engenders higher population level susceptibility in the summer baseline season. This increased population level susceptibility, perhaps combined with higher humidity levels, allows for increased influenza activity in the summer. This would be in contrast to drier subregions in the US where influenza may transmit more efficiently during the winter and decrease the susceptible population to lower levels thereby limiting significant summer influenza activity. If this is indeed the case, the increased summer influenza activity and higher levels of susceptibility in the southeastern US may be related to recent observations that influenza epidemics often begin in the southeastern US [21, 32] including the 2009 A/H1N1 fall pandemic wave [33]. It is not readily clear how this dynamic would progress over multiple years, but previous studies have shown that environmental factors may have effects on influenza dynamics that emerge in the subsequent years [34].

A recent study by Dalziel et al. (2018) showed a strong relationship between influenza-like-illness (ILI), humidity and population density [35]. The study suggests that humidity affects the intensity of epidemics, but that this effect is diminished in large cities due to increased transmission efficiency in crowded conditions. Interestingly, we did not find that population was a strong predictor of cross-seasonal ratios in our analysis which conflicts with their results. This may indicate that cross-seasonal ratios are not a strong measure of epidemic intensity, or that this relationships was obscured when we aggregated the data to a subregional scale (this was necessary to increase the number of observations in each unit). That said, our conclusions regarding humidity are consistent with their findings, i.e., influenza cases are distributed more evenly across the year in more humid areas. We were also able to show evidence of substantial differences between the cross-seasonal patterning of influenza A and B across the US, which Dalziel et al. were unable to discern because their study used ILI data. Further, our analysis suggests the possibility that summer humidity may also play a role which Dalziel et al. did not consider.

We did not have sufficient data in many populations in the western US to include in the analysis and, thus, we were unable to make any general conclusions about cross-seasonal influenza activity in these populations. As more data become available in future years it will be interesting to assess cross-seasonal influenza activity in these regions. For example, many areas in California are characterized by absolute humidity levels during the influenza season that are commensurate with humidity levels in much of the southeastern US. Other regions such as the high elevation areas and deserts of the interior western US may also have unusual seasonal characteristics given their long low-humidity seasons. For instance, the influenza season in Phoenix, AZ during the 2016–2017 season persisted into June, substantially later than other subregions.

This study had several significant limitations. First, only two years of data were available which is problematic for generalizing our findings beyond the years evaluated. However, given that the data included information about influenza types A and B, we were able to assess patterns across 4 distinct influenza seasons, including the severe 2017–2018 A/H3N2 season, and the broad consistency of our results provides evidence that the patterns observed are a stable feature of influenza activity in the US. Another major limitation inherent to this dataset is that we do not know if protocols and practices of the health care organizations and clinics administering tests vary seasonally. We assumed that the probability that a patient with ILI symptoms was administered an influenza test was time invariant (or temporal variations were relatively homogeneous across subregions), as differences in these practices could have significant consequences for our findings. That said, although variability of clinical practices are likely present in the data, these effects cannot easily explain the regional patterns of cross-seasonal influenza activity observed since the data in each region were generated by numerous clinicians at multiple independent healthcare organizations.

Our analysis shows that cross-seasonal influenza activity varies across the US and that this variation is spatially structured. We suggest that the variability of cross-seasonal influenza activity may be the result of decreased influenza activity during the influenza season (~September-May), and increased activity during the summer baseline season (~June-August) in the southeastern US and Hawaii. We also showed that variability in cross-seasonal influenza activity is significantly associated with absolute humidity during the influenza season, and we found some evidence that humidity during the summer may also play a role. The differences in the seasonal nature of influenza across regions may highlight important differences in influenza dynamics across the US.

## Supporting information

S1 File Supplementary results. Excel spreadsheet with the raw results for each influenza year, influenza type and subregion.
(CSV)

S2 File Raw data. A CSV file that contains the raw data used in this analysis.
(CSV)

S1 Fig Population and subregions. Top map shows the total population of each subregion. The markers represent reporting sensors for both years. The dark areas indicate areas with a high density of sensors. The bottom map shows the subregions in relation to states for comparison.
(TIFF)

S2 Fig Maps from sensitivity analysis. Maps of cross-seasonal ratios for each subregion by influenza year and type for 180-day and 330-day influenza seasons. Plus/minus symbols indicate subregions where the ratio was significantly below/above expected value (based on Fisher’s exact tests). Dashed areas are subregions with no or inadequate numbers of positive tests.
(TIFF)

S3 Fig Percent positive during influenza season. Percent-positivity rates during the epidemic seasons for influenza A and B during 2016–2017 and 2017–2018. Plus/minus symbols indicate subregions where the ratio was significantly below/above expected value (based on Fisher’s exact tests). Dashed areas are subregions with no or inadequate numbers of positive tests.
(TIFF)

S4 Fig Subregional humidity. Weighted average and max specific humidity levels by subregions for the epidemic and baseline season for 2016–2017 and 2017–2018.
(TIFF)

S1 Table Bivariate results for sensitivity analysis (180-days). Results of bivariate mixed-effects regression analysis where the cross-seasonal ratio was the dependent variable and the influenza season was defined as the 180 days with the maximum number of cases. The models were sorted in ascending order by AIC.
(DOCX)

S2 Table Bivariate results for sensitivity analysis (330-days). Results of bivariate mixed effects regression analysis where the cross-seasonal ratio was the dependent variable and the influenza season was defined as the 330 days with the maximum number of cases.
(DOCX)

S3 Table Multivariate results for sensitivity analysis (180-days). Results of select multivariate mixed-effects regression models where the log cross-seasonal ratio was the dependent variable and the influenza season was defined as the 180 days with the maximum number of cases. The models were sorted in ascending order by AIC. A null model with dummy variables was included for comparison.
(DOCX)

S4 Table Multivariate results for sensitivity analysis (330-days). Results of select multivariate mixed-effects regression models where the log cross-seasonal ratio was the dependent variable and the influenza season was defined as the 330 days with the maximum number of cases. The models were sorted in ascending order by AIC. A null model with dummy variables was included for comparison.
(DOCX)
