# Supplementary figures and images for: Seasonal characteristics of influenza vary regionally across US
Source: PLoS One. 2019 Mar 6;14(3):e0212511. doi: 10.1371/journal.pone.0212511 (PMC6402651; doi:10.1371/journal.pone.0212511)

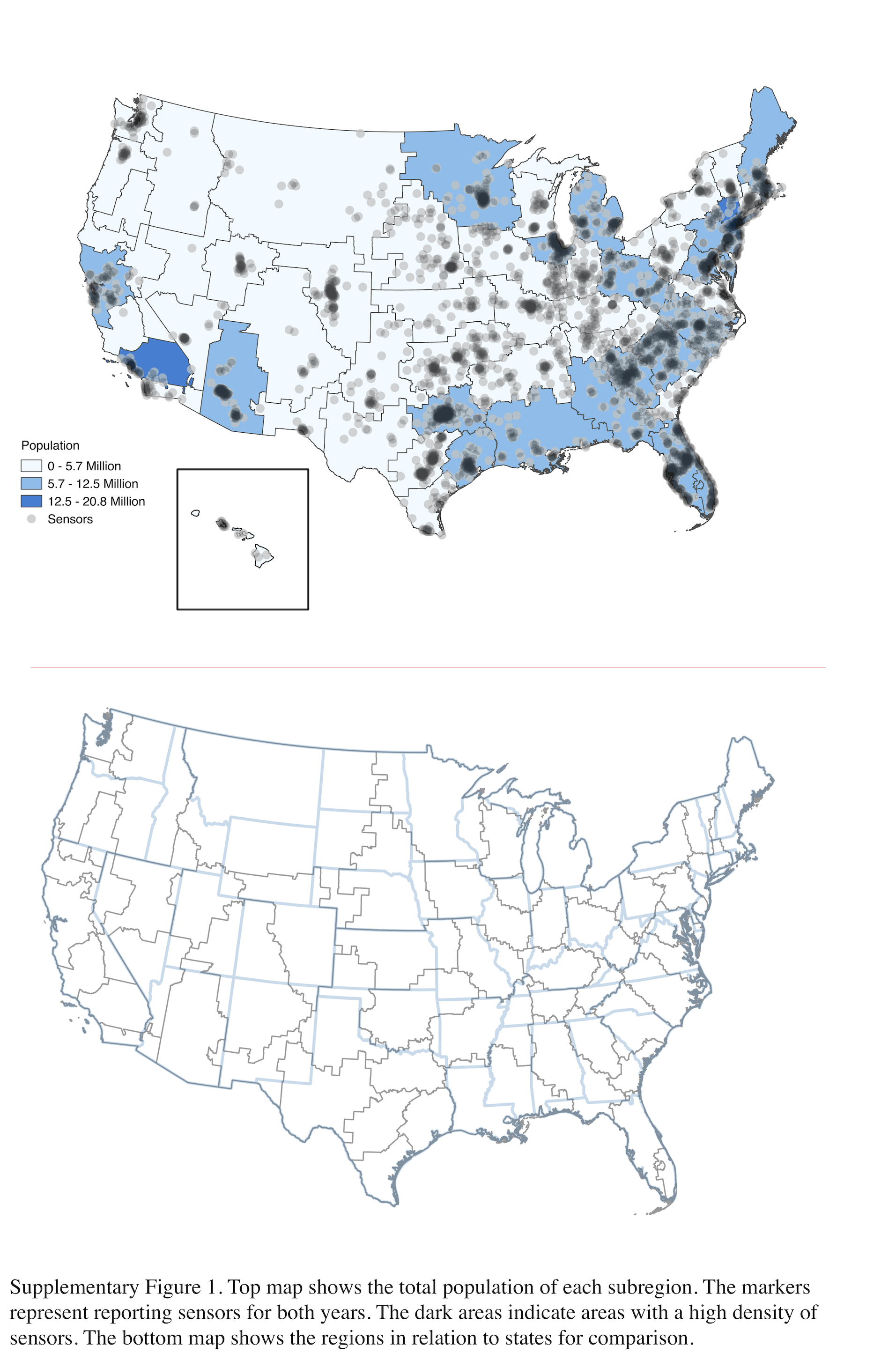

Supplement: S1 Fig — Top map shows the total population of each subregion. The markers represent reporting sensors for both years. The dark areas indicate areas with a high density of sensors. The bottom map shows the subregions in relation to states for comparison. (TIFF) [file pone.0212511.s003.tiff]

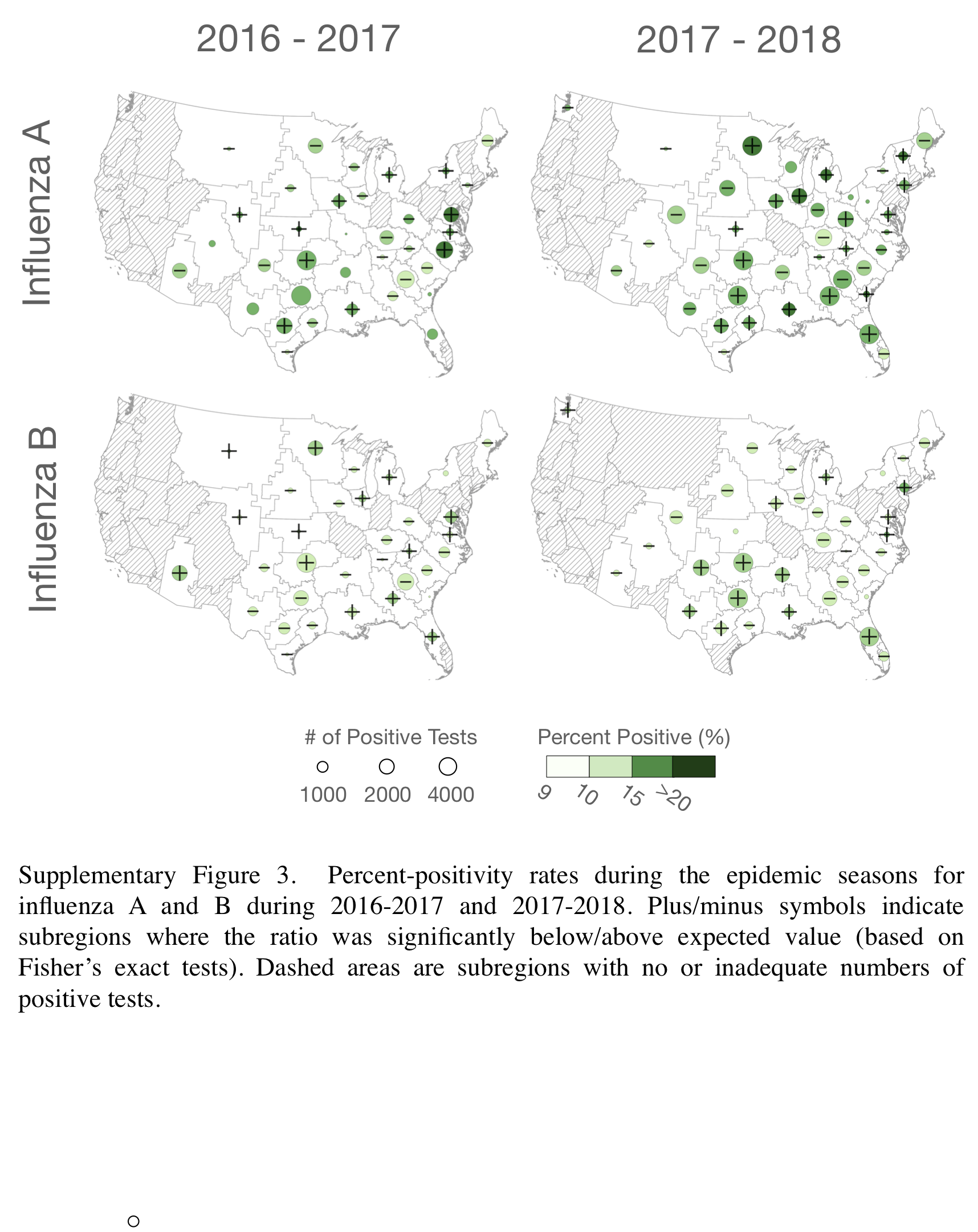

Supplement: S3 Fig — Percent-positivity rates during the epidemic seasons for influenza A and B during 2016–2017 and 2017–2018. Plus/minus symbols indicate subregions where the ratio was significantly below/above expected value (based on Fisher’s exact tests). Dashed areas are subregions with no or inadequate numbers of positive tests. (TIFF) [file pone.0212511.s005.tiff]

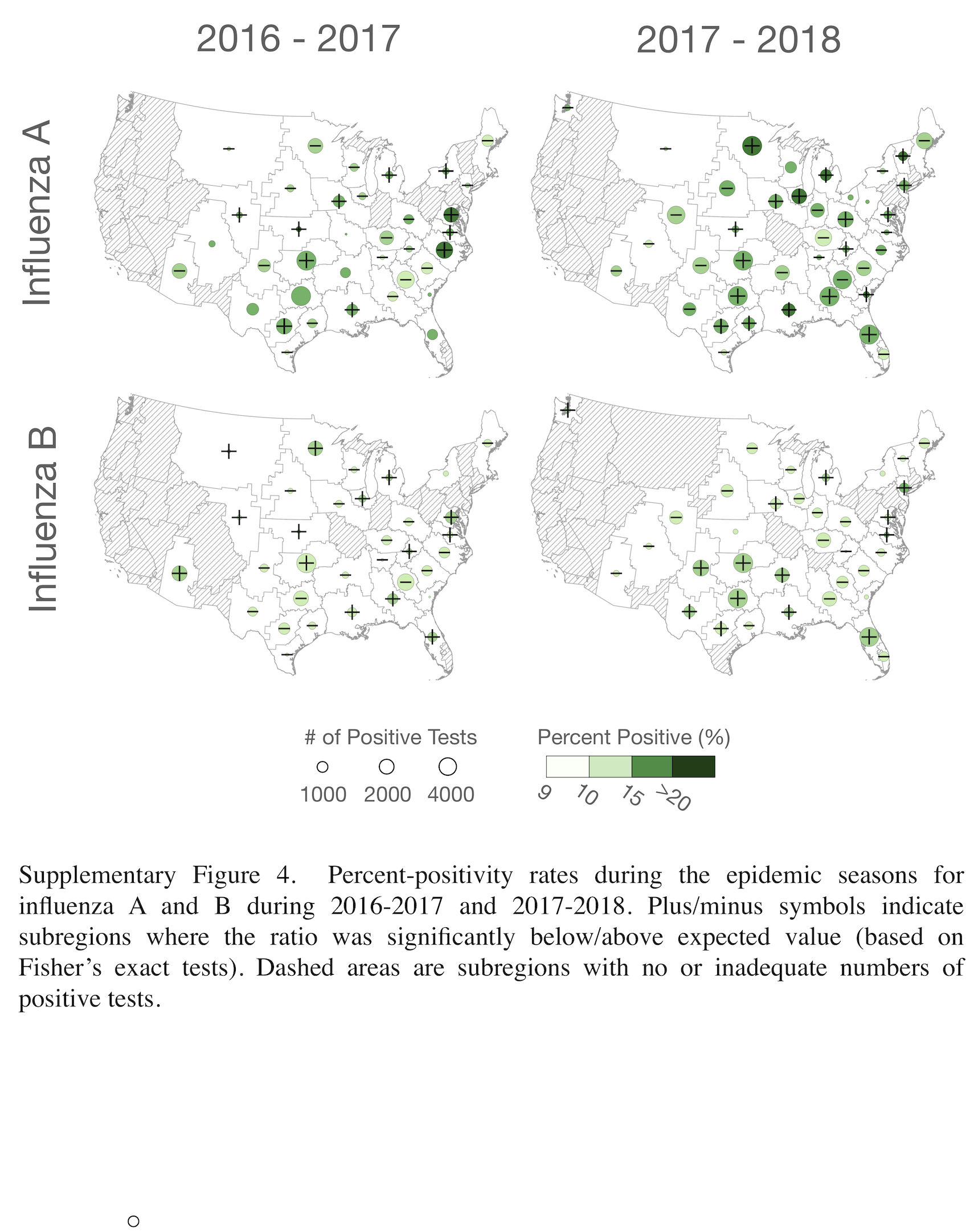

Supplement: S4 Fig — Weighted average and max specific humidity levels by subregions for the epidemic and baseline season for 2016–2017 and 2017–2018. (TIFF) [file pone.0212511.s006.tiff]
