# Supplementary material for: Seasonal characteristics of influenza vary regionally across US
Source: PLoS One. 2019 Mar 6;14(3):e0212511. doi: 10.1371/journal.pone.0212511 (PMC6402651; doi:10.1371/journal.pone.0212511)
Supplement: S1 Table — Results of bivariate mixed-effects regression analysis where the cross-seasonal ratio was the dependent variable and the influenza season was defined as the 180 days with the maximum number of cases. The models were sorted in ascending order by AIC. (DOCX) [file pone.0212511.s007.docx]

| Predictors | Bivariate | |
| --- | --- | --- |
|  | Coefficients  (95% CI) | AIC/BIC |
| Weighted specific humidity  (influenza season) | -0.11  ( -0.13, -0.09) | 86/98 |
| Weighted temperature  (influenza season) | -0.04  (-.04, -.03) | 91/103 |
| Weighted specific humidity  (baseline season) | -0.06  (-0.08, -0.04) | 132/144 |
| Latitude | 0.03  (0.03, 0.06) | 133/146 |
| Weighted temperature  (baseline season) | -0.04  (-0.05, -0.02) | 138/150 |
| Vaccination Rate | 0.03  (0.00, 0.06) | 156/168 |
| Longitude | 0.00  (-0.00, 0.01) | 159/171 |
| Total Population / 10^6^ | 0.00  (-0.02, 0.03) | 160/173 |

**S1_Table**. **Bivariate Results for Sensitivity Analysis (180-days)**. Results of bivariate mixed-effects regression analysis where the cross-seasonal ratio was the dependent variable and the influenza season was defined as the 180 days with the maximum number of cases. The models were sorted in ascending order by AIC.
