# Supplementary material for: Seasonal characteristics of influenza vary regionally across US
Source: PLoS One. 2019 Mar 6;14(3):e0212511. doi: 10.1371/journal.pone.0212511 (PMC6402651; doi:10.1371/journal.pone.0212511)
Supplement: S2 Table — Results of bivariate mixed effects regression analysis where the cross-seasonal ratio was the dependent variable and the influenza season was defined as the 330 days with the maximum number of cases. (DOCX) [file pone.0212511.s008.docx]

| Predictors | Bivariate | |
| --- | --- | --- |
|  | Coefficients  (95% CI) | AIC/BIC |
| Weighted specific humidity  (baseline season) | -0.04  (-0.04, -0.03) | 152/164 |
| Weighted temperature  (baseline season) | -0.02  (-0.03, -0.02) | 155/167 |
| Weighted specific humidity  (influenza season) | -0.08  ( -0.05, -0.10) | 190/202 |
| Weighted temperature  (influenza season) | -0.03  (-.04, -.02) | 198/210 |
| Longitude | 0.01  (0.00, 0.01) | 214/226 |
| Total Population / 10^6^ | 0.03  (0.00, 0.05) | 214/227 |
| Latitude | 0.01  (-0.00, 0.03) | 216/228 |
| Vaccination Rate | -0.00  (-0.03, 0.03) | 219/231 |

**S2_Table.** Results of bivariate mixed-effects regression analysis where the cross-seasonal ratio was the dependent variable and the influenza season was defined as the 330 days with the maximum number of cases.
