# Supplementary material for: Seasonal characteristics of influenza vary regionally across US
Source: PLoS One. 2019 Mar 6;14(3):e0212511. doi: 10.1371/journal.pone.0212511 (PMC6402651; doi:10.1371/journal.pone.0212511)
Supplement: S3 Table — Results of select multivariate mixed-effects regression models where the log cross-seasonal ratio was the dependent variable and the influenza season was defined as the 180 days with the maximum number of cases. The models were sorted in ascending order by AIC. A null model with dummy variables was included for comparison. (DOCX) [file pone.0212511.s009.docx]

|  | Model 1 | | Model 2 | | Model 3 | | Null Model | |
| --- | --- | --- | --- | --- | --- | --- | --- | --- |
| Predictors | Coefficients  (95% CI) | AIC/BIC | Coefficients  (95% CI) | AIC/BIC | Coefficients  (95% CI) | AIC/BIC | Coefficients  (95% CI) | AIC/BIC |
| Latitude | — | 33/54 | — | 35/59 | — | 44/66 | — | 62/80 |
| Longitude | — |  | — |  | — |  | — |  |
| Weighted specific humidity  (influenza season) | -0.08  (-0.10, -0.06) |  | -0.08  (-0.11, -0.04) |  | — |  | — |  |
| Weighted specific humidity  (baseline season) | — |  | -0.01  (-0.03, 0.02) |  | — |  | — |  |
| Weighted temperature  (influenza season) | — |  | — |  | -0.03  (-0.03, -0.02) |  | — |  |
| Weighted temperature  (baseline season) | — |  | — |  | — |  | — |  |
| Total Population | — |  | — |  | — |  | — |  |
| Vaccination Rate | — |  | — |  | — |  | — |  |
| Influenza A/2016-2017 | 0.09  (-0.01, 0.20) |  | 0.09  (-0.01, 0.20) |  | -0.15  (-0.29, -0.01) |  | 0.00  (-0.10, 0.10) |  |
| Influenza A/2017-2018 | 0.24  (0.14, 0.33) |  | 0.24  (0.14, 0.34) |  | 0.14  (0.01, 0.27) |  | 0.24  (0.14, 0.34) |  |
| Influenza B/2016-2017 | -0.23  (-0.33, -0.12) |  | -0.23  (-0.34, -0.12) |  | -0.40  (-0.56, -0.25) |  | -0.37  (-0.47, -0.26) |  |
| Influenza B/2017-2018 | 0 |  | 0 |  | 0 |  | 0 |  |

**S3_Table**. Results of select multivariate mixed-effects regression models where the log cross-seasonal ratio was the dependent variable and the influenza season was defined as the 180 days with the maximum number of cases. The models were sorted in ascending order by AIC. A null model with dummy variables was included for comparison.
